# Adherence to the EAT-Lancet Diet Among Urban and Rural Latin American Adolescents: Associations with Micronutrient Intake and Ultra-Processed Food Consumption

**DOI:** 10.3390/nu17122048

**Published:** 2025-06-19

**Authors:** Rulamán Vargas-Quesada, Rafael Monge-Rojas, Sonia Rodríguez-Ramírez, Jacqueline Araneda-Flores, Leandro Teixeira Cacau, Gustavo Cediel, Diego Gaitán-Charry, Tito Pizarro Quevedo, Anna Christina Pinheiro Fernandes, Alicia Rovirosa, Tania G. Sánchez-Pimienta, María Elisa Zapata

**Affiliations:** 1 Nutrition and Health Unit, Costa Rican Institute for Research and Education on Nutrition and Health (INCIENSA), Ministry of Health, Tres Ríos 42250, Costa Rica; rmonge@inciensa.sa.cr; 2 Nutrition and Health Research Center (CINyS), National Institute of Public Health, Mexico (INSP), Cuernavaca 62100, Mexico; scrodrig@insp.mx; 3 Departamento de Nutrición y Salud Pública, Facultad de Ciencias de la Salud y de los Alimentos, Universidad del Bío-Bío, Avda. Collao 1202 Casilla 5-C, Concepción 4051381, Chile; jaraneda@ubiobio.cl; 4 Department of Nutrition, School of Public Health, University of São Paulo, São Paulo 01246-904, Brazil; lcacau@usp.br; 5 Grupo de Investigación Saberes Alimentarios (SAL), Universidad de Antioquia UdeA, Calle 70 No. 52-21, Medellín 050010, Colombia; gustavo.cedielg@udea.edu.co (G.C.); diego.gaitan@udea.edu.co (D.G.-C.); 6 Facultad de Ciencias Médicas, Universidad de Santiago de Chile, Av. Libertador Bernardo O’Higgins 3363, Estación Central 9170022, Chile; tito.pizarro@usach.cl; 7 Carrera de Nutrición y Dietética, Facultad de Medicina-Clínica Alemana, Universidad del Desarrollo, Santiago 7610658, Chile; apinheiro@udd.cl; 8 Centro de Estudios Sobre Nutrición Infantil “Dr. Alejandro O’Donnell” (CESNI), Ciudad Autónoma de Buenos Aires C1072AAF, Argentina; arovirosa@cesni.org.ar (A.R.); mezapata@cesni.org.ar (M.E.Z.); 9 National Council of Humanities, Sciences and Technologies (CONAHCYT), Nutrition and Health Research Center (CINyS), National Institute of Public Health, Mexico (INSP), Cuernavaca 62100, Mexico; taniagsp@hotmail.com

**Keywords:** EAT-Lancet diet, adolescents, Latin America, micronutrient intake, ultra-processed foods

## Abstract

**Background/Objectives:** Adolescents in Latin America are experiencing rising rates of overweight/obesity and non-communicable diseases, while public health nutrition efforts targeting this group remain limited. This study explores adherence to the EAT-Lancet diet and its relationship with micronutrient adequacy and ultra-processed food (UPF) consumption. **Methods:** Cross-sectional data from national nutrition surveys of 19,601 adolescents across six Latin American countries were analyzed. Data on sociodemographics, anthropometrics, and dietary habits were collected using standardized questionnaires and 24 h dietary recalls or food records. Nutrient intake was estimated via statistical modeling, and nutrient adequacy ratios were based on age- and sex-specific requirements. UPF intake was classified using the NOVA system, and adherence to the EAT-Lancet diet was assessed with the Planetary Health Diet Index. **Results:** Overall adherence to the EAT-Lancet diet was low (mean score: 28.3%). Rural adolescents had higher adherence than urban adolescents, and those aged 10–13 and 17–19 showed better adherence compared to adolescents aged 14–16. Adolescents from lower socioeconomic backgrounds adhered more than those from higher socioeconomic backgrounds. Adherence varied from 20.2% in Argentina to 30.2% in Brazil and Chile. Higher adherence was associated with lower UPF intake. Among urban adolescents, greater adherence was linked to a higher risk of inadequate riboflavin, niacin, and cobalamin intake, a trend not observed in rural adolescents. **Conclusions:** Adherence to the EAT-Lancet diet is low among Latin American adolescents, particularly in urban areas. Public health efforts should prioritize reducing UPF consumption, improving access to nutrient-dense, culturally appropriate foods, and supporting fortified staple foods.

## 1. Introduction

Adolescence has often been considered the healthiest stage of life, partly due to adolescents’ historically low use of health services [1]. However, this assumption has been inaccurate since the 1960s, yet its persistence continues to render adolescents nearly invisible in health policies [1]. Alarmingly, a systematic review based on data from 2008 to 2013 estimated that between 16.5 and 22.1 million adolescents in Latin America were living with excess weight (overweight or obesity), with prevalence rates ranging from 16.7% in Colombia to 35.0% in Mexico [2]. However, the most recent data available in the region reveal a clear upward trend, with reported prevalence reaching 18% in Colombia [3], 25% in Brazil [3], 27% in Argentina [4], 30.9% in Costa Rica [5], 32.8% in Chile [6], and 41% in Mexico [3]. Additionally, approximately 35% are affected by metabolic syndrome [7,8], highlighting the urgent need for effective non-communicable disease (NCD) prevention strategies. Despite this growing public health concern, adolescents remain largely overlooked in government nutrition programs [9,10,11,12], even as global data show increasing rates of excess weight and related comorbidities in this age group [10,13].

Adopting the predominantly plant-based diet recommended by the EAT-Lancet Commission could be an effective strategy to reduce the risk of NCDs from adolescence through adulthood while simultaneously lowering the environmental impact of food production in Latin America [14]. The EAT-Lancet diet emphasizes the consumption of fruits, vegetables, legumes, whole grains, nuts, seeds, and unsaturated oils. It also recommends a low to moderate intake of dairy, fish, seafood, and poultry, while advocating minimal to no consumption of red and processed meats, animal fats, and added sugars [14]. Unlike other plant-based diets, the EAT-Lancet diet emphasizes not only a high intake of plant-based foods but also a broader dietary framework designed to optimize human health while minimizing environmental impact [14].

This approach is particularly important given the significant transformations in Latin America’s food systems over recent decades [15]. Traditional diets, once based on fresh foods, have increasingly been replaced by ultra-processed foods (UPFs), high in sugar, fat, and sodium, from the early stages of childhood [3,15,16], posing a serious public health concern for adolescents in the region [17]. Urbanization, globalization, and aggressive marketing have been key drivers of this shift, contributing to the rising prevalence of NCDs, such as obesity, diabetes, and cardiovascular diseases [3,15]. Reversing this trend requires improving access to nutritious foods and promoting healthier dietary patterns [18]. Adolescence represents a critical window of opportunity where optimizing health and well-being can serve as a “circuit breaker” for the intergenerational transmission of NCD risk [19].

Several studies in adults from developed countries have shown that adherence to the EAT-Lancet pattern is associated with a lower risk of obesity and diet-related diseases, as well as benefits for metabolic, cardiovascular, and cognitive health, and a reduced risk of cancer [20,21,22,23,24,25,26]. While the EAT-Lancet diet offers potential benefits for NCD prevention from an early age, its adequacy in providing sufficient micronutrient intake remains debated. Some studies suggest that greater adherence to this diet leads to higher micronutrient intake [27,28,29], while others show the opposite [30,31]. This issue is particularly relevant from a public health perspective when promoting the EAT-Lancet pattern in adolescents, as adequate micronutrient intake is essential for growth and sexual maturation, and the body’s ability to retain these nutrients increases substantially during the growth spurt [32].

Research on adolescent adherence to the EAT-Lancet diet is limited and primarily focuses on urban populations, despite evidence suggesting that rural adolescents may adhere more closely to healthier diets [33,34,35,36]. Studies have reported low adherence to the EAT-Lancet diet among adolescents, with rates as low as 29.1% in Brazil [37] and 29.9% in Europe [29], highlighting a significant gap between current dietary patterns and the recommended healthy and sustainable diet. Currently, data on this topic are scarce in Latin America [37]. Therefore, this study aimed to assess adherence to the EAT-Lancet diet among urban and rural adolescents from six Latin American countries and explore its associations with micronutrient intake adequacy and energy intake from UPFs.

## 2. Materials and Methods

### 2.1. Sample and Setting

This cross-sectional study utilized baseline data from various representative nutrition surveys conducted in six Latin American countries over the last 15 years. The study sample included 19,601 urban and rural adolescents from the following surveys: the Argentina National Survey of Nutrition and Health (2018–2019; *n* = 2310, included only urban adolescents) [38], the Brazilian National Dietary Survey (2017–2018; *n* = 8475) [39,40], the Chile National Food Consumption Survey (2009–2010; *n* = 746) [41], the National Survey of Nutritional Situation in Colombia (2015; *n* = 6027) [42], the Study of Eating Habits in Adolescents in Costa Rica (2017; *n* = 818) [43], and the Mexico National Health and Nutrition Survey Midway (2016; *n* = 1225) [44]. The main methodological characteristics of each survey conducted in six Latin American countries are summarized below.

In Argentina, the National Survey of Nutrition and Health (2018–2019) used a stratified probabilistic cross-sectional design with national coverage, targeting children under 5, women aged 10 to 49, and individuals aged 10 and older. Data were collected through face-to-face interviews, 24-hour dietary recalls (24-HRs), and anthropometric measurements [38]. The Brazilian National Dietary Survey (2017–2018) applied a complex sampling design with national and regional representation, collecting data from approximately 57,920 households on income, expenditures, food consumption through 24-HRs, and anthropometry, among other variables [39,40]. The Chile National Food Consumption Survey (2009–2010) assessed around 5000 individuals aged 2 to 65 from diverse geographical areas through 24-HRs, food frequency questionnaires, and anthropometric evaluations [41]. Colombia’s National Survey of Nutritional Situation (2015) included a population-based sample of over 44,000 households of all ages, employing a stratified probabilistic design to gather information on health, diet through 24-HRs, physical activity, and biochemical markers [42]. The Study of Eating Habits in Adolescents in Costa Rica (2017) focused on youth aged 13 to 19, employing a multistage regional sampling method and collecting data through 3-day food records (3-FRs), anthropometry, and assessments of psychosocial influences on eating behavior [43]. In Mexico, the National Health and Nutrition Survey Midway (2016) used a multistage stratified cluster sampling strategy to assess 29,795 individuals from 9474 households across three age groups. Data were obtained using standardized tools on housing, chronic disease, physical activity, and dietary assessment using 24-HRs, alongside anthropometric and biochemical measures to evaluate conditions such as obesity, hypertension, and diabetes [45].

Collectively, these surveys share key methodological elements, including probabilistic cross-sectional designs, validated dietary assessment methods, and standardized anthropometric measurements. These commonalities facilitate regional comparisons of dietary patterns and nutritional status, despite variations in survey periods, target populations, and sample sizes.

Each survey was approved by the local ethics committees in their respective countries prior to data collection, ensuring compliance with informed consent and assent procedures for participants, except in Brazil, where the survey is publicly available and did not require ethics committee approval [39,40]. Additionally, the Scientific Ethics Committee of the Costa Rican Institute for Research and Education in Nutrition and Health (INCIENSA) approved this study’s overarching protocol on 2 October 2024 (INCIENSA-CEC-of-2024-61).

### 2.2. Demographic and Socioeconomic Status Variables

Sex, age, and socioeconomic status (SES) were assessed using a country-specific questionnaire designed to comply with national legislative standards or recognized local formats. SES was classified as low, middle, or high based on each country’s national indices. The area of residence was determined according to national standards and categorized as urban or rural [38,39,40,41,42,43,44]. Since the data were collected from surveys conducted over different years, standardizing the measurement of SES or ensuring a consistent classification of the area of residence across the participating countries was not feasible for this study, since it used secondary datasets. However, the definitions used, although not homogeneous, are based on conceptual frameworks recognized in each data source, which ensures sufficient internal consistency for analytical purposes.

### 2.3. Anthropometric Assessment

Height and weight were measured by trained nutritionists following standardized procedures in each country [46] except in Brazil, where these anthropometric measures were auto-reported [39,40]. Before being weighed, adolescents were instructed to empty their pockets, remove their shoes, and take off any excess outerwear. Body mass index (BMI) was calculated using measured height and weight values based on the standard formula: weight (kg)/height (m^2^). Body weight status was classified based on the BMI Z-score for age, following the World Health Organization guidelines [47]: <−2: underweight; ≥−2 and <+1: healthy weight (eutrophy); ≥+1 and <+2: overweight; and ≥+2: obesity. For practical interpretation, the underweight and healthy weight categories were combined into a non-overweight/obesity group, while the overweight and obesity categories were merged into a single group.

### 2.4. Dietary Assessment

Various methods were employed to capture information on actual food intake in order to determine food consumption. These included two complete 24-hour recalls (24-HRs) in Brazil, one 24-HR on the first day and a subsample for the second day in Argentina, Chile, Colombia, and Mexico, following the recommendations of Deitchler et al. [48], and a 3-day food record (3-FR) in Costa Rica. Although two different methods were used, this does not greatly affect the comparability of the results, since both approaches record actual intake and are therefore suitable for estimating absolute—rather than relative—intakes of energy and other food components. Additionally, both methods provide high specificity in describing foods and preparation methods, as well as flexibility in analyzing intake data [49].

A data cleaning process was conducted on each country’s dataset to identify dietary intake outliers prior to this secondary analysis, as documented in the methodology of each respective survey [38,39,40,41,42,43,44], ensuring the use of clean datasets. The food composition databases used to convert food weights into energy and nutrient intake were those commonly employed in each country. This approach allowed for the inclusion of both typical local foods and those mandatorily fortified with micronutrients, in accordance with each nation’s specific fortification policies [38,39,40,41,42,43,44], thereby maximizing the accuracy of nutrient intake estimates within each country’s context and minimizing the risk of under- or overestimation of intake and adequacy.

### 2.5. Usual Dietary Intake

Usual intake of energy and 13 micronutrients—including thiamin (vitamin B1), riboflavin (vitamin B2), niacin (vitamin B3), pyridoxine (vitamin B6), folate equivalents (vitamin B9), cobalamin (vitamin B12), vitamin C, vitamin A, vitamin D, calcium, iron, magnesium, and zinc—was estimated using intake data derived from available 24-HRs or 3-FRs. The dataset from Argentina included all of these micronutrients except pyridoxine, while the dataset from Colombia contained information on only five micronutrients: vitamin C, vitamin A, calcium, iron, and zinc.

To estimate the usual intake, the Multiple Source Method (MSM) was employed in Argentina, Brazil, Chile, Costa Rica, and Mexico [50]. MSM is a modeling technique designed to correct for within-person variability in dietary intake data by combining information from repeated 24-HRs or FRs with covariate information, thereby producing more reliable estimates of long-term (usual) micronutrient intake at the individual level [51]. In Colombia, usual nutrient intake was assessed using the PC Software for Intake Distribution Estimation (PC-SIDE, version 1.0) [52], in accordance with national standards. PC-SIDE applies a similar adjustment for intra-individual variation and is specifically designed to estimate the distribution of usual intakes across all individuals in the sample, facilitating comparison to micronutrient reference values [51]. Both methods ensure accurate and representative intake assessments, which are critical for evaluating micronutrient adequacy and informing public health nutrition policies.

### 2.6. Nutrient Adequacy

The Nutrient Adequacy Ratio (NAR) for 13 micronutrients was calculated by comparing each participant’s usual intake to the Estimated Average Requirement (EAR) based on their respective sex and age group, following the guidelines established by the National Academy of Medicine of the United States [53]. Consequently, Equation (1) was used [54]:

(1) $$NAR=\left(\frac{{Usual\;dietary\;intake}_{nutrient}}{{EAR}_{nutrient}}\right)$$

The NAR for iron was calculated assuming a bioavailability of 10%, in accordance with the World Health Organization’s guidelines for diets in developing countries [55]. Nutrient adequacy for a specific nutrient was defined as follows: adequate intake indicated by a NAR value ≥ 1, and inadequate intake indicated by a NAR value < 1.

### 2.7. Ultra-Processed Foods Intake

Ultra-processed foods (UPFs) were classified using the NOVA classification system, following an iterative process applied in similar studies [56]. NOVA food classification was conducted independently by research teams in each country, who triangulated information to resolve discrepancies and reach a consensus. The classification included four categories: (1) unprocessed or minimally processed foods, (2) processed culinary ingredients, (3) processed foods, and (4) UPFs [57,58]. Information on homemade preparations in each database was broken down into individual ingredients, based on recipes provided by adolescents during food consumption data collection. For each adolescent, energy intake from UPFs on the first day of the 24-HR (or the second day of the 3-FR in Costa Rica) was used to calculate their individual caloric share from UPFs (%TEI from UPFs).

### 2.8. The Planetary Health Diet Index

The Planetary Health Diet Index (PHDI) was used to assess adherence to the EAT-Lancet diet. This index includes all food groups defined by the EAT-Lancet and employs a gradual scoring system based on consumption levels [59]. PHDI scores were calculated using an energy intake ratio, determined by dividing the energy from all foods within each PHDI component by the total daily energy intake from all foods consumed, excluding alcoholic beverages [60]. For each adolescent, energy intake from each PHDI component on the first day of the 24-HR (or the second day of the 3-FR in Costa Rica) was used to calculate their individual PHDI score.

The PHDI consists of 16 components categorized into four groups: adequacy (nuts and peanuts, fruits, legumes, vegetables, whole grain cereals), optimum (eggs, dairy products, fish and seafood, tubers and potatoes, vegetable oils), ratio (dark green vegetables/total vegetables, red-orange vegetables/total vegetables), and moderation (red meat, poultry, animal fats, added sugars). Details on PHDI components, scoring criteria, and cutoff points based on the EAT-Lancet diet are described elsewhere [60] and presented in the Supplementary Materials (Table S1).

All consumed foods and mixed dishes, including recipes, were disaggregated to the ingredient level before classifying each food item into the 16 PHDI components, following the methodology proposed by Cacau et al. for extracting PHDI components from food consumption data [60]. Disaggregation allowed for accurate classification of the energy contribution of each food item according to its corresponding PHDI component. UPFs were disaggregated to estimate their content of added sugars, vegetable oils, and animal (saturated) fats. This careful process ensured accurate categorization of these three components within the PHDI, preventing any under- or overestimation.

To illustrate the relative contribution of each component, the authors established classification criteria: high relative contribution (≥50% of available points per component), intermediate relative contribution (20–49% of available points per component), and low relative contribution (<20% of available points per component). These thresholds were defined to facilitate comparisons among PHDI components. The total PHDI score ranges from 0 to 150, with higher scores reflecting greater adherence to the EAT-Lancet diet. Relative adherence to the reference diet (%) is calculated by dividing the PHDI score by 150 points and then multiplying the result by 100.

### 2.9. Statistical Analyses

Data analyses accounted for sample complexity using survey weights. Continuous variables were reported as means with their respective 95% confidence interval (95% CI), while categorical variables were reported as frequencies (%). PHDI scores and total energy intake from UPFs were compared across categories of sex, age group, SES, area, country, and weight status using the adjusted-Wald test (ANOVA-like) for complex survey data [61]. Additionally, differences among categories were considered statistically significant when the 95% CIs did not overlap.

Multivariate Poisson regression models with robust variance were employed to assess the association between relative adherence to the EAT-Lancet diet (PHDI quintiles) and inadequate intake of 13 micronutrients based on the EAR. This method was preferred over logistic regression because it produces estimates with narrower confidence intervals and mitigates the common problem of overestimating risk associated with odds ratios in logistic regression, particularly in studies with a cross-sectional design and for outcomes with high prevalence (≥10%) [62,63,64,65,66].

Generalized linear regression models (GLMs) with gamma distribution and Log link function were utilized to evaluate the association between relative adherence to the EAT-Lancet diet (PHDI quintiles) and energy intake from UPFs. The gamma distribution with the Log link function was preferred since energy intakes from UPFs are strictly positive values [67].

The association trend was assessed using orthogonal polynomial contrasts for linear trend among the PHDI quintiles within each model (Poisson models for inadequate nutrient intakes and GLMs for energy intake from UPFs). This approach helps prevent misinterpretation of the dependent variable’s behavior in intermediate quintiles and confirms a linear trend across all quintiles. Non-multicollinearity within each model was evaluated using the variance inflation factor, and over-dispersion in Poisson models was assessed using negative binomial models. Additionally, stratified analyses by area and age group were conducted for both types of Poisson models and GLMs to evaluate potential confounding effects of area in the multi-adjusted models.

All tests were two-tailed, with *p*-values < 0.05 considered statistically significant. Data analyses were conducted using Stata^®^ 14.1 (StataCorp LLC, College Station, TX, USA), incorporating the “survey” command to account for sample complexity.

## 3. Results

### 3.1. Characteristics, Planetary Health Diet Index, and Ultra-Processed Food Intake of Study Participants

The average age of the study population was 14.9 (SE = 0.06) years. Most of the study population lived in urban areas (80.5%) and were not overweight or obese (65.7%). The proportions for sex, age groups, and SES categories were similar within the study population (Table 1).

The mean PHDI score of the study population was 42.4 points (95% CI: 42.0–42.8) out of 150, representing relative adherence to the EAT-Lancet diet of 28.3%. The PHDI score was significantly higher among rural adolescents, compared to their urban counterparts (*p* < 0.05). Also, the score was significantly higher in adolescents aged 10–13 years (early adolescence) compared to those aged 14–16 years (middle adolescence), but similar to the score of adolescents aged 17–19 years (late adolescence). Similarly, in terms of SES, the score was significantly higher in adolescents with middle SES compared to those with high SES, but similar to the score of individuals with low SES (*p* < 0.05). There were no significant differences according to sex or weight status (*p* > 0.05) (Table 1).

Significant differences in PHDI scores were observed among countries, ranging from 30.3 points (95% CI: 29.6–31.0) in Argentina to 45.3 points (95% CI: 44.2–46.5) in Chile (*p* < 0.05). Brazil had similar PHDI scores than Chile (45.1 points, 95% CI: 44.8–45.5), followed by Costa Rica (42.6 points, 95% CI: 41.8–43.5), Mexico (40.4 points, 95% CI: 39.3–41.5), and Colombia (35.6 points, 95% CI: 35.2–36.0) (Table 1).

The mean energy intake from UPFs in the study population was 28.5% (95% CI: 27.8–29.2). Energy intake from UPFs was significantly higher among individuals from urban areas and those with high SES (*p* < 0.05). No significant differences were observed among participants according to their sex, age group, or weight status (*p* > 0.05).

Significant differences in energy intake from UPFs were observed across countries, ranging from 21.9% (95% CI: 21.3–22.6) in Colombia to 36.9% (95% CI: 35.4–38.4) in Costa Rica. Chile and Brazil had similar energy intake from UPFs (26.7%, 95% CI: 24.5–28.9 and 26.5%, 95% CI: 25.8–27.2, respectively), followed by Argentina (29.6%, 95% CI: 28.2–30.9) and Mexico (33.4%, 95% CI: 31.7–35.3) (*p* < 0.05).

### 3.2. Planetary Health Diet Index Components Score

**Figure 1:**
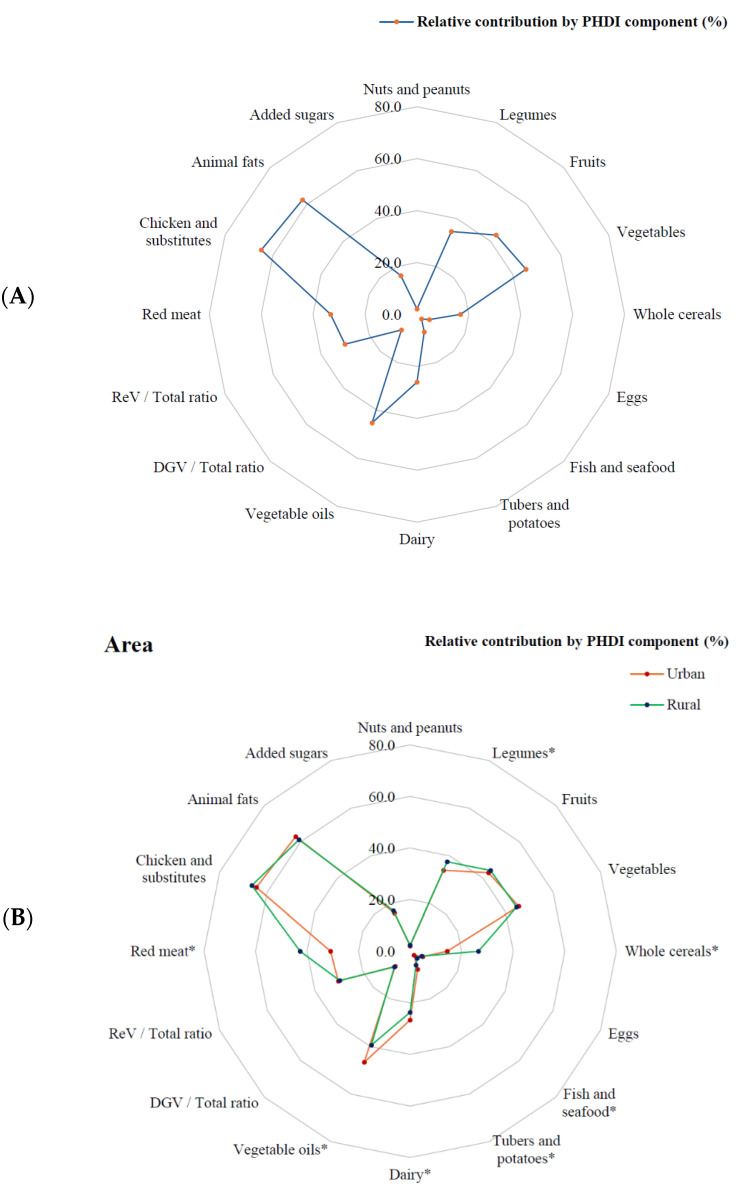
Mean relative contribution ^1^ of each Planetary Health Diet Index component among adolescents in the study (**A**) and according to their area of residence (**B**). PHDI: Planetary Health Diet Index; DGV: dark green leafy vegetables; ReV: red and orange vegetables. * PHDI components with significant differences between urban and rural areas (*p* < 0.05). Argentina does not include rural participants. ^1^ Determined using survey weights.

The overall descriptive analysis of PHDI component scores showed the mean relative contribution of each component to the total PHDI score based on their available points (Figure 1A). Some components had a high relative contribution (≥50% of available points per component) to adherence, such as chicken and substitutes (64.9%) and animal fats (62.4%). Others showed an intermediate relative contribution (20–49%), including vegetables (45.5%), vegetable oils (45.2%), fruits (43.2%), legumes (34.6%), red meat (33.2%), the ReV/total ratio (red and orange vegetables ratio) (30.0%), and dairy (26.1%). The remaining PHDI components had a low relative contribution (<20%) to adherence (Figure 1A).

When analyzing the relative contribution of PHDI components by area of residence, rural adolescents had significantly higher relative contributions than their urban counterparts in several components, including red meat (42.7% vs. 30.9%), legumes (37.5% vs. 33.9%), whole cereals (26.5% vs. 14.4%), and fish and seafood (3.8% vs. 2.1%) (*p* < 0.05) (Figure 1B).

In contrast, urban adolescents had significantly higher relative contributions in vegetable oils (46.6% vs. 39.4%), dairy (26.7% vs. 23.7%), and tubers and potatoes (7.6% vs. 5.8%) (*p* < 0.05). There were no significant differences between urban and rural areas for chicken and substitutes, animal fats, vegetables, fruits, the ReV/total ratio, added sugars, DGV/total ratio (dark green leafy vegetables ratio), eggs, and nuts and peanuts (*p* > 0.05) (Figure 1B).

### 3.3. Association Between Adherence to the Planetary Health Diet Index and Nutrient Intakes

After adjusting for sex, age, SES, area, and country, adolescents with high adherence to the EAT-Lancet diet (5th PHDI quintile) had significantly higher risks of nutrient inadequate intake for riboflavin (33.7%), niacin (49.9%), and cobalamin (30.2%) compared to those with low adherence (1st quintile) (*p*-trend < 0.05) (Table 2). Conversely, high adherence was associated with significantly lower risks of inadequate intake for folate equivalents (32.6%), vitamin C (40.3%), and magnesium (28.0%) (*p*-trend < 0.05). No significant association was observed between high adherence to the PHDI and the inadequate intake of thiamin, pyridoxine, vitamin A, vitamin D, calcium, iron, or zinc (*p*-trend > 0.05) (Table 2).

An additional stratified analysis by area revealed some differences compared to the area-adjusted model. Among urban adolescents, those with high adherence to the EAT-Lancet diet had significantly higher risks of nutrient inadequacy for riboflavin (40.3%), niacin (13.2%) and cobalamin (39.6%) compared to those with low adherence (*p*-trend < 0.05). In contrast, among rural adolescents, significant associations between adherence to the dietary pattern and higher risk of nutrient inadequacy were not observed (*p*-trend < 0.05) (Table 3).

Conversely, high adherence among urban adolescents was associated with significantly lower risks of inadequate intake for folate equivalents (30.9%), vitamin C (42.3%), and magnesium (24.7%) (*p*-trend < 0.05). Among rural adolescents, significant associations between adherence to the dietary pattern and lower risk of nutrient inadequacy were observed for the same three nutrients: folate equivalents (33.2%), vitamin C (22.5%), and magnesium (39.9%) (*p*-trend < 0.05) (Table 3).

### 3.4. Association Between Adherence to the EAT-Lancet Diet and Ultra-Processed Foods Intake

A significant negative association was observed between adherence to the EAT-Lancet diet and energy intake from UPFs. In the fully adjusted model, adolescents with high adherence to the EAT-Lancet diet (5th PHDI quintile) had significantly lower energy intake from UPFs (β= −0.573, 95% CI: −0.638 to −0.507, *p*-trend < 0.05), compared to those with low adherence (1st quintile) (Table 4).

A stratified analysis by area yielded results consistent with the fully adjusted model (urban: β = −0.545, 95% CI: −0.620 to −0.471, *p*-trend < 0.05; rural: β = −0.646, 95% CI: −0.802 to −0.489, *p*-trend < 0.05) (Table 4). Supplementary Figure S1 shows the PHDI quintile predictive margins for the models presented in Table 4 (Figure S1). A similar trend was observed in the PHDI quintile predictive margins when analyzed across three adolescent age groups: early adolescence (10–13 years), middle adolescence (14–16 years), and late adolescence (17–19 years) (Figure S2).

## 4. Discussion

This study examines adherence to the EAT-Lancet diet among urban and rural adolescents in six Latin American countries, analyzing its association with adequate micronutrient intake and UPF consumption. The findings reveal a low adherence rate of 28.3% among Latin American adolescents, consistent with trends observed in their European counterparts [29], and is slightly below the 29.5% reported among urban adults in eight countries across the Latin American region [68].

Our results highlight the need for significant investments in transforming Latin American food systems to ensure the successful adoption of a sustainable dietary pattern from adolescence. This transformation is particularly critical in urban areas, where adolescents demonstrate significantly lower adherence to the EAT-Lancet diet compared to their rural counterparts. This disparity may be influenced by the characteristics of the urban food system, which provides a wide variety and increased availability of processed, prepared, and UPFs, as well as increased accessibility to animal-source foods [69,70]. Taken together, these characteristics of the urban food system may potentially lead to deviations from the EAT-Lancet diet.

Conversely, rural adolescents tend to follow a more plant-based diet, which aligns more closely with the principles of the EAT-Lancet diet. This dietary pattern may be influenced by the lower purchasing power of rural households compared to urban ones [69,71], limiting the consumption of animal products while promoting a higher intake of legumes—an affordable food—as well as whole cereals (e.g., corn and corn tortillas), fish, and seafood, likely sourced through traditional subsistence agriculture or local food production [72,73]. Furthermore, the lower energy intake from UPFs among rural adolescents helps preserve traditional diets, which are characterized by minimally processed foods and freshly prepared dishes, preventing their displacement by the unhealthy “Westernized” dietary pattern observed in urban areas [16,74]. This is particularly relevant in a public health context, given a recent analysis of a nationwide population-based study in Brazil, which shows a dose–response inverse association between UPF consumption and adherence to a healthy and sustainable diet [75].

While greater adherence to the EAT-Lancet diet among adolescents may reduce the risk of inadequacies in folate equivalents, vitamin C, and magnesium, it concurrently appears to increase the likelihood of insufficient intakes of certain B vitamins, including riboflavin, niacin, and cobalamin. This elevated risk was statistically significant among urban adolescents but not among their rural counterparts. Although the current analysis does not yield a fully substantiated explanation, some plausible hypotheses—grounded in existing knowledge of dietary patterns and food systems in Latin America—may help contextualize this finding. One potential explanation is that urban adolescents adhering more closely to the EAT-Lancet dietary pattern may consume fewer animal-source foods, such as milk, eggs, and meat, which constitute the primary natural sources of these B vitamins. Since the EAT-Lancet framework promotes a predominantly plant-based diet with limited animal products, close adherence may inadvertently reduce intakes of these essential micronutrients, even when overall dietary quality improves as indicated by adherence scores. Additionally, this discrepancy may partially reflect differences in the consumption of fortified staple foods. Many Latin American countries have established public health policies mandating fortification of products such as wheat flour, corn flour, and rice with B vitamins. However, urban adolescents may consume these staples less frequently—possibly due to a dietary shift away from traditional foods toward ultra-processed products, which are often unfortified, or due to evolving food preferences influenced by contemporary urban food environments [15,16].

Given that this study did not aim to identify specific food sources contributing to micronutrient intake, the analysis is limited in its ability to elucidate the dietary pathways underlying the observed divergence in B vitamin inadequacies between urban and rural adolescents. To address this gap, future research should incorporate detailed, food-level dietary intake data to more accurately characterize the underlying factors in adolescents’ diets that contribute to these micronutrient disparities.

Nevertheless, the identified differences in the risk of inadequate B vitamin intake between urban and rural adolescents contribute to the ongoing discussions on the association between adherence to the EAT-Lancet diet and micronutrient intakes, demonstrating that it may have positive effects on some nutrients while potentially having negative effects on others. The absence of statistically significant associations between adherence to the EAT-Lancet diet and some vitamins and minerals may be attributed to multiple factors. First, for certain nutrients—such as folate equivalents, iron, and zinc—baseline intake levels among adolescents may have been sufficient regardless of EAT-Lancet adherence, likely due to national food fortification policies or the routine consumption of staple foods rich in these nutrients, such as pulses. Second, low variability in the intake of some micronutrients across adherence levels may have limited the ability to detect associations. This is especially relevant for nutrients commonly derived from fortified or staple foods, which may contribute to more uniform intake levels across the population. Third, differences in nutrient bioavailability may also play a role. Although plant-based diets often provide nutrients with lower bioavailability [76], this may not have translated into measurable inadequacy for certain micronutrients—such as iron and magnesium—particularly within the constraints of cross-sectional data. Finally, dietary diversity within adherence categories may contribute to heterogeneous micronutrient profiles. Even among individuals with similar EAT-Lancet adherence scores, the types and combinations of foods consumed can differ markedly, leading to non-uniform patterns of nutrient intake. Despite these possible hypotheses, further research is needed to explore the factors contributing to the inadequate intakes of B vitamins in adolescents with high adherence to the EAT-Lancet diet.

From a public health perspective, inadequate B vitamin intake is particularly concerning, as the EAT-Lancet diet discourages the consumption of refined foods, which in Latin America are a key source of mandatory fortification with essential nutrients, including B vitamins [77]. While whole grains can provide these vitamins in high concentrations [78], the consumption of these foods is not a common part of Latin American food culture [79]. Although the EAT-Lancet Commission recommends adapting the proposed dietary framework to diverse sociocultural contexts, it does not provide alternatives for substituting whole grains with refined grains [14], even though the greenhouse gas footprint is similar between both types of grains [80,81].

Additionally, greater adherence to the EAT-Lancet diet among urban adolescents increases the risk of cobalamin inadequate intake, which can lead to adverse health outcomes [82]. Studies have shown that the EAT-Lancet diet and other plant-based dietary patterns are associated with cobalamin inadequacy due to their emphasis on reducing animal product consumption [30,83,84], the primary source of this nutrient [85].

Due to the approach of the EAT-Lancet diet, particularly its limitations on refined foods and animal products, it is essential to explore strategies that support a sustainable food system in the Latin American context, which meets the high nutritional needs during adolescence while also mitigating the negative health effects of UPF consumption from an early age [86,87,88,89]. As the EAT-Lancet diet is a global recommendation, strategies for promoting a healthy and sustainable diet must consider the cultural diversity of Latin American adolescents’ eating habits and align with local food traditions for successful adoption [90]. A key challenge is ensuring adolescents’ willingness to modify their eating habits, as cultural traditions, perceptions, attitudes, and beliefs play a significant role in shaping new dietary behaviors and fostering the adoption of a sustainable diet [90].

A critical factor for policymakers in promoting a healthy and sustainable diet is reducing UPF consumption. Our results show that adherence to the EAT-Lancet dietary pattern declines across all stages of adolescence (early, middle, and late) as UPF consumption increases, with adherence being lowest in middle adolescence. This could be due to greater susceptibility to peer social norms and the adoption of unhealthy eating behaviors [91,92], as normative social influence on eating is both potent and pervasive [93]. However, a more thorough investigation is needed to gain a clearer understanding of the factors driving this trend.

On the other hand, increasing adolescents’ adherence to a healthy and sustainable diet requires considering the high cost and disparities in healthy eating across the Americas region. According to the FAO’s report, “Cost and Affordability of a Healthy Diet (CoAHD)”, the cost of a healthy diet in Chile is 4.54 USD PPP (dollar per person per day), while in Brazil it is 4.45 USD PPP and in the United States of America 2.63 USD PPP [94]. Therefore, adherence to a healthy and sustainable diet is heavily influenced by access to affordable healthy food, which presents a significant challenge for countries in implementing or reformulating public policies to create healthier environments.

This study has some limitations that should be considered. First, the dietary surveys used are not recent (2009–2019) and were all conducted before the COVID-19 pandemic. As a result, current dietary habits among adolescents may differ from those recorded. Nevertheless, these surveys remain the most recent and only available data for each participating country. The lack of updated dietary surveys is a persistent issue across Latin America [95], with most data relying on Household Consumption and Expenditures Surveys, which provide household-level consumption data rather than precise individual estimates [96]. Second, the classification of adolescents’ residential areas as urban or rural and their households’ SES was not standardized, as it followed each country’s official methodology. However, the observed trends in EAT-Lancet diet adherence and energy intake from UPFs by area remained consistent despite the different classifications. Third, the findings of this study should be interpreted in light of its cross-sectional design, which limits the analysis to associations and does not permit causal inferences between EAT-Lancet diet adherence, micronutrient adequacy, and UPF intake. Moreover, due to the use of secondary datasets, important potentially confounding variables—such as food access, education, and local dietary patterns—were not available for inclusion in the analysis. Fourth, anthropometric measures in the Brazilian survey were self-reported, which could lead to an underestimation of the proportion of the study participants with excess weight. Fifth, this study did not account for stratification or clustering details in the survey-weight analysis; however, using only weights can provide valid estimates, as stratification and clustering primarily affect the sampling variability of the estimates rather than the parameter estimates [97]. This study also has notable strengths. First, the methodology for determining the PHDI, classifying UPFs, and assessing micronutrient intake adequacy was standardized across all countries. Additionally, we employed the Planetary Health Diet Index, which was validated in previous studies, to evaluate adherence to the EAT-Lancet diet [60]. Second, data analyses accounted for sample complexity using survey-weighted procedures. This approach ensured that the analysis accurately reflects the diverse population represented in the sample, mitigating potential biases and enhancing the robustness of the findings [98]. By incorporating these advanced statistical techniques, the study improved the generalizability and precision of the results, providing a more reliable foundation for drawing conclusions [99].

## 5. Conclusions

To our knowledge, this study is the first to examine the adherence of Latin American adolescents to the EAT-Lancet diet, offering valuable insights for public health decision-makers. The study’s conclusions highlight the low adherence to the EAT-Lancet diet among adolescents in Latin America, especially in urban areas. Urban adolescents show lower adherence, likely due to higher consumption of UPFs and animal-based foods. In contrast, rural adolescents tend to follow more plant-based diets, better aligned with the principles of the EAT-Lancet diet, as they consume more legumes, whole grains, and fish and seafood, while limiting their intake of other animal products and UPFs. However, greater adherence to the EAT-Lancet diet increases the risk of inadequate intake of B vitamins, such as riboflavin, niacin, and cobalamin, particularly among urban adolescents. The underlying reasons for this remain unclear. This study underscores the need for strategies that adapt healthy and sustainable diets to the Latin American context, considering local food traditions and the cultural diversity of adolescents in the region. Ensuring adolescents’ willingness to modify their eating habits is essential for driving a shift toward a healthy and sustainable diet within this population group.

## Figures and Tables

**Table 1:** Study population characteristics, PHDI score, and energy intake from UPFs among subgroups of adolescents in the study.

Characteristic	Total	PHDI Score (Points)			UPF (%TEI)		
	% ^1^	Mean	95% CI	*p*-Value ^2^	Mean	95% CI	*p*-Value ^2^
Overall	100.0	42.4	42.0–42.8	-	28.5	27.8–29.2	-
Sex							
Male	50.9	42.0	41.4–42.7	0.050	28.4	27.4–29.3	0.773
Female	49.1	42.9	42.3–43.4		28.6	27.6–29.6	
Age							
10–13 years	32.2	43.5 ^a^	42.8–44.2	0.001	28.0	27.2–28.9	0.548
14–16 years	35.3	41.6 ^b^	41.0–42.2		28.8	27.5–30.0	
17–19 years	32.5	42.3 ^ab^	41.5–43.1		28.7	27.3–30.2	
Country							
Argentina	5.4	30.3 ^a^	29.6–31.0	<0.001	29.6 ^a^	28.2–30.9	<0.001
Brazil	55.4	45.1 ^b^	44.8–45.5		26.5 ^b^	25.8–27.2	
Chile	4.2	45.3 ^b^	44.2–46.5		26.7 ^b^	24.5–28.9	
Colombia	5.4	35.6 ^c^	35.2–36.0		21.9 ^c^	21.3–22.6	
Costa Rica	0.2	42.6 ^d^	41.8–43.5		36.9 ^d^	35.4–38.4	
Mexico	29.4	40.4 ^e^	39.3–41.5		33.4 ^e^	31.7–35.3	
Area							
Urban	80.5	42.1	41.6–42.6	<0.001	29.8	29.0–30.6	<0.001
Rural ^3^	19.5	43.9	43.2–44.6		23.0	21.8–24.3	
Socioeconomic status							
Low	30.3	42.4 ^ab^	41.8–43.1	0.029	22.1 ^a^	21.2–23.1	<0.001
Middle	33.5	43.1 ^b^	42.5–43.7		28.3 ^b^	27.4–29.3	
High	36.2	41.8 ^a^	41.0–42.4		34.0 ^c^	32.6–35.4	
Weight status ^4^							
Non-overweight/obesity	65.7	42.5	42.0–43.0	0.668	28.4	27.4–29.3	0.589
Overweight/obesity	34.3	42.3	41.6–43.0		28.8	27.6–29.9	

^1^ Determined using survey weights. ^2^ *p*-value corresponds to the adjusted-Wald test (ANOVA-like) comparing groups. Mean values within the same variable that do not share a common letter are significantly different (*p* < 0.05). ^3^ Argentina does not include rural participants. ^4^ Weight status according to BMI-for-age categories. PHDI: Planetary Health Diet Index; CI: confidence interval; UPF: ultra-processed food; %TEI: percentage of total energy intake.

**Table 2:** Association between high adherence to the PHDI and micronutrient inadequate intake among adolescents in the study.

Nutrient	PR ^1^	95% CI	*p*-Value ^2^	*p*-Trend ^3^
Vitamins				
Thiamin ^4^	1.052	0.911–1.215	0.485	0.733
Riboflavin ^4^	1.337	1.119–1.599	0.001	0.006
Niacin ^4^	1.499	1.250–1.798	<0.001	<0.001
Pyridoxine ^4,5^	1.026	0.919–1.146	0.648	0.692
Folate equivalents ^4^	0.674	0.596–0.763	<0.001	<0.001
Cobalamin ^4^	1.302	1.024–1.656	0.032	0.041
Vitamin C	0.597	0.536–0.666	<0.001	<0.001
Vitamin A	1.001	0.937–1.069	0.982	0.762
Vitamin D ^4^	0.998	0.996–1.001	0.180	0.652
Minerals				
Calcium	1.016	0.957–1.079	0.604	0.941
Iron	0.993	0.908–1.086	0.882	0.485
Magnesium ^4^	0.720	0.663–0.783	<0.001	<0.001
Zinc	1.041	0.888–1.219	0.623	0.739

Multivariate Poisson regression analysis with robust variance estimation: ^1^ Prevalence ratio of nutrient inadequacy comparing the 5th PHDI quintile to the 1st quintile (reference group); adjusted for sex, age, SES, area, and country. Nutrient inadequate intake was defined as a Nutrient Adequacy Ratio (NAR) < 1. ^2^ *p*-value corresponds to the Wald test for the 5th PHDI quintile as a category of the PHDI within each model. ^3^ *p*-value for trend corresponds to the linear trend among PHDI quintiles within each model. ^4^ Nutrient not available in the Colombia dataset. ^5^ Nutrient not available in the Argentina dataset. PHDI quintiles: min–max score/1st: 0.7–29.6; 2nd: 29.7–36.8; 3rd: 36.9–43.3; 4th: 43.4–50.8; 5th: 50.9–89.8. PHDI: Planetary Health Diet Index.

**Table 3:** Association between high adherence to the PHDI and micronutrient inadequate intake among adolescents in the study, according to their area of residence.

Nutrient	Urban				Rural ^6^			
	Inadequate Intake				Inadequate Intake			
	PR ^1^	95% CI	*p*-Value ^2^	*p*-Trend ^3^	PR ^1^	95% CI	*p*-Value ^2^	*p*-Trend ^3^
Vitamins								
Thiamin ^4^	1.058	0.882–1.268	0.541	0.734	0.949	0.749–1.201	0.662	0.835
Riboflavin ^4^	1.403	1.111–1.772	0.004	0.024	1.032	0.760–1.401	0.842	0.355
Niacin ^4^	1.432	1.154–1.778	0.001	0.002	1.251	0.797–1.963	0.331	0.155
Pyridoxine ^4,5^	1.089	0.941–1.262	0.252	0.356	0.943	0.798–1.115	0.494	0.532
Folate equivalents ^4^	0.691	0.594–0.805	<0.001	<0.001	0.668	0.559–0.799	<0.001	<0.001
Cobalamin ^4^	1.396	1.009–1.934	0.044	0.043	1.039	0.755–1.428	0.974	0.800
Vitamin C	0.577	0.508–0.654	<0.001	<0.001	0.775	0.630–0.953	0.016	0.005
Vitamin A	0.989	0.911–1.073	0.785	0.701	1.004	0.922–1.095	0.913	0.915
Vitamin D ^4^	1.000	0.997–1.002	0.729	0.944	0.997	0.991–1.004	0.419	0.177
Minerals								
Calcium	1.021	0.948–1.099	0.587	0.806	0.959	0.878–1.047	0.350	0.481
Iron	1.005	0.899–1.123	0.936	0.730	0.904	0.813–1.004	0.059	0.078
Magnesium ^4^	0.753	0.683–0.830	<0.001	<0.001	0.601	0.515–0.702	<0.001	<0.001
Zinc	1.094	0.906–1.320	0.352	0.955	0.877	0.672–1.146	0.337	0.254

Multivariate Poisson regression analysis with robust variance estimation: ^1^ Prevalence ratio of nutrient inadequacy comparing the 5th PHDI quintile to the 1st quintile (reference group); adjusted for sex, age, SES, and country. Nutrient inadequate intake was defined as a Nutrient Adequacy Ratio (NAR) < 1. ^2^ *p*-value corresponds to the Wald test for the 5th PHDI quintile as a category of the PHDI within each model. ^3^ *p*-value for trend corresponds to the linear trend among PHDI quintiles within each model. ^4^ Nutrient not available in the Colombia dataset. ^5^ Nutrient not available in the Argentina dataset. ^6^ Argentina does not include rural participants. PHDI quintiles urban: min–max score/1st: 0.7–28.6; 2nd: 28.7–35.9; 3rd: 36.0–42.4; 4th: 42.5–50.1; 5th: 50.2–89.8. PHDI quintiles rural: min–max score/1st: 5.4–32.2; 2nd: 32.3–39.8; 3rd: 39.9–45.9; 4th: 46.0–52.7; 5th: 52.8–89.4. PHDI: Planetary Health Diet Index.

**Table 4:** Association between adherence to the PHDI and energy intake from UPFs among adolescents in the study.

Models		PHDI Quintile	UPF (%TEI)			
			β ^1^	95% CI	*p*-Value ^5^	*p*-Trend ^6^
Fully-adjusted ^2^ (*n* = 19,601)		1st	ref	ref	-	<0.001
		2nd	−0.089	−0.155–−0.023	0.008	
		3rd	−0.214	−0.279–−0.149	<0.001	
		4th	−0.309	−0.370–−0.247	<0.001	
		5th	−0.573	−0.638–−0.507	<0.001	
Stratified analysis	Urban ^3^ (*n* = 14,671)	1st	ref	ref	-	<0.001
		2nd	−0.083	−0.158–−0.008	0.031	
		3rd	−0.199	−0.275–−0.124	<0.001	
		4th	−0.301	−0.373–−0.229	<0.001	
		5th	−0.545	−0.620–−0.471	<0.001	
	Rural ^3,4^ (*n* = 4930)	1st	ref	ref	-	<0.001
		2nd	−0.031	−0.176–0.115	0.681	
		3rd	−0.235	−0.361–−0.109	<0.001	
		4th	−0.336	−0.471–−0.202	<0.001	
		5th	−0.646	−0.802–−0.489	<0.001	

Generalized linear regression model: ^1^ Coefficient of %TEI from UPFs for each PHDI quintile. ^2^ Adjusted for sex, age, SES, area, and country (gamma distribution with Log link function). ^3^ Stratified analysis by area: adjusted for sex, age, SES, and country (gamma distribution with Log link function). ^4^ Argentina does not include rural participants. ^5^ *p*-value corresponds to the Wald test for each PHDI quintile within each model. ^6^ *p*-value for trend corresponds to the linear trend among PHDI quintiles within each model. PHDI quintiles: min–max score/1st: 0.7–29.6; 2nd: 29.7–36.8; 3rd: 36.9–43.3; 4th: 43.4–50.8; 5th: 50.9–89.8. PHDI quintiles urban: min–max score/1st: 0.7–28.6; 2nd: 28.7–35.9; 3rd: 36.0–42.4; 4th: 42.5–50.1; 5th: 50.2–89.8. PHDI quintiles rural: min–max score/1st: 5.4–32.2; 2nd: 32.3–39.8; 3rd: 39.9–45.9; 4th: 46.0–52.7; 5th: 52.8–89.4. PHDI: Planetary Health Diet Index; %TEI: percentage of total energy intake; UPFs: ultra-processed foods.

## Data Availability

The data that support the findings of this study are available from the corresponding author upon reasonable request. The data are not publicly available due to privacy reasons.

## Supplementary Materials

The following supporting information can be downloaded at https://www.mdpi.com/article/10.3390/nu17122048/s1, Table S1. Planetary Health Diet Index components and food groups, cutoff points for scoring, and corresponding point values; Figure S1. Energy intake from UPF predictive margins by PHDI quintile according to area; Figure S2. Energy intake from UPF predictive margins by PHDI quintile according to age group.

## Abbreviations

The following abbreviations are used in this manuscript:

24-HR	24-hour recall
3-FR	3-day food record
CI	Confidence interval
DGV	Dark green leafy vegetables
EAR	Estimated Average Requirement
GLM	Generalized linear regression model
MSM	Multiple Source Method
NAR	Nutrient Adequacy Ratio
NCD	Non-communicable disease
PC-SIDE	PC Software for Intake Distribution Estimation
PHDI	Planetary Health Diet Index
PR	Prevalence ratio
ReV	Red and orange vegetables
SES	Socioeconomic status
UPF	Ultra-processed food
