# Supplementary material for: Adherence to the EAT-Lancet Diet Among Urban and Rural Latin American Adolescents: Associations with Micronutrient Intake and Ultra-Processed Food Consumption
Source: Nutrients. 2025 Jun 19;17(12):2048. doi: 10.3390/nu17122048 (PMC12195673; doi:10.3390/nu17122048)
Supplement: Supplementary file 1 [file nutrients-17-02048-s001.zip › nutrients-3685536-supplementary.pdf]

Adherence to the EAT-Lancet Diet Among Urban and Rural Latin American Adolescents: Associations with Micronutrient Intake and Ultra-Processed Food Consumption

Supplementary materials

Table S1. Planetary Health Diet Index components and food groups, cutoff points for scoring, and corresponding point values.

Components / Food groups	Score (points) [a]				
	0	5	10	5	0
**Adequacy component**			**Energy density (%TEI)**		
Nuts and peanuts	0.0	↔	≥11.6		
Legumes [b]	0.0	↔	≥11.3		
Fruits	0.0	↔	≥5.0		
Vegetables	0.0	↔	≥3.1		
Whole grains	0.0	↔	≥32.4		
**Optimum component**					
Eggs	0.0	↔	0.8	↔	≥1.5
Fish and seafood	0.0	↔	1.6	↔	≥5.7
Tubers and potatoes	0.0	↔	1.6	↔	≥3.1
Dairy [c]	0.0	↔	6.1	↔	≥12.2
Vegetables oils [d]	0.0	↔	16.5	↔	≥30.7
**Ratio component**					
DGV/total vegetables [e]	0.0 ↔	29.5		29.5 ↔	100.0
ReV/total vegetables [f]	0.0 ↔	38.5		38.5 ↔	100.0
**Moderation component**					
Red meat [g]	≥2.4	↔	0.0		
Chicken and substitutes	≥5.0	↔	0.0		
Animal fats [h]	≥1.4	↔	0.0		
Added sugars	≥4.8	↔	0.0		

[a] All table values are expressed as energy densities of the EAT-Lancet diet (%TEI). [b] Includes soy. [c] Excludes dairy fats. [d] Includes palm oil. [e] Ratio of energy intake from dark green leafy vegetables to total vegetables. [f] Ratio of energy intake from red and orange vegetables to total vegetables. [g] Includes beef, lamb, and pork. [h] Includes lard, tallow, and dairy fats. Adapted from Cacau *et al.*, 2021 (REF).

%TEI: percentage of total energy intake; DGV: dark green leafy vegetables; ReV: red and orange vegetables.

Panel A

Fully-adjusted[1]

Panel B

Urban[2]

Panel C

Rural[2,3]

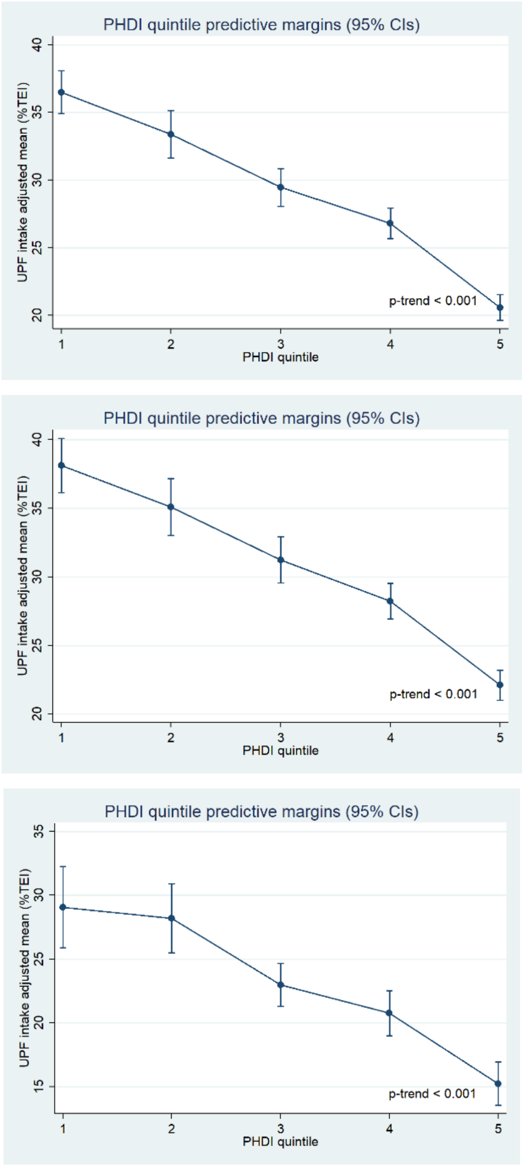

Figure S1. Energy intake from UPF predictive margins by PHDI quintile according to area.

Generalized linear regression model: [1]Panel A: adjusted for sex, age, SES, area, and country. [2]Stratified analysis by area: adjusted for sex, age, SES, and country (Panel B: urban; Panel C: rural). [3]Argentina does not include rural participants. %TEI: percentage of total energy intake.

PHDI quintiles: min–max score / 1st: 0.7–29.6; 2nd: 29.7–36.8; 3rd: 36.9–43.3; 4th: 43.4–50.8; 5th: 50.9–89.8.

PHDI quintiles urban: min–max score / 1st: 0.7–28.6; 2nd: 28.7–35.9; 3rd: 36.0–42.4; 4th: 42.5–50.1; 5th: 50.2–89.8.

PHDI quintiles rural: min–max score / 1st: 5.4–32.2; 2nd: 32.3–39.8; 3rd: 39.9–45.9; 4th: 46.0–52.7; 5th: 52.8–89.4.

Panel A

10-13 years

Panel B

14-16 years

Panel C

17-19 years

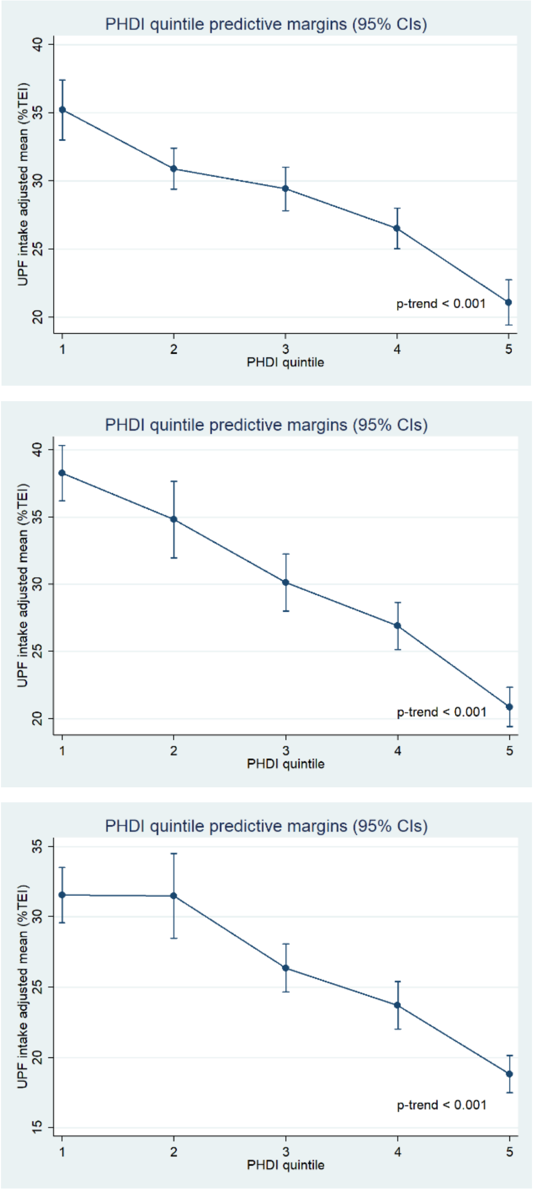

Figure S2. Energy intake from UPF predictive margins by PHDI quintile according to age group.

Generalized linear regression model with stratified analysis by age group: adjusted for sex, age, SES, area and country (Panel A: 10-13 years; Panel B: 14-16 years; Panel C: 17-19 years). %TEI: percentage of total energy intake.

PHDI quintiles 10-13 years: min–max score / 1st: 5.0–30.8; 2nd: 30.9–38.5; 3rd: 38.6–44.8; 4th: 44.9–52.2; 5th: 52.3–83.0.

PHDI quintiles 14-16 years: min–max score / 1st: 1.1–28.1; 2nd: 28.2–35.0; 3rd: 35.1–41.7; 4th: 41.8–49.3; 5th: 49.4–89.8.

PHDI quintiles 17-19 years: min–max score / 1st: 0.7–30.5; 2nd: 30.6–38.3; 3rd: 38.4–44.5; 4th: 44.6–51.9; 5th: 52.0–85.8.
